# Inhaler Devices for Delivery of LABA/LAMA Fixed-Dose Combinations in Patients with COPD

**DOI:** 10.1007/s41030-019-0090-1

**Published:** 2019-03-13

**Authors:** Anthony D’Urzo, Kenneth R. Chapman, James F. Donohue, Peter Kardos, M. Reza Maleki-Yazdi, David Price

**Affiliations:** 1 Department of Family and Community Medicine, University of Toronto, Toronto, ON Canada; 2 Asthma and Airway Centre, University Health Network, University of Toronto, Toronto, ON Canada; 3 Pulmonary Diseases and Critical Care Medicine, Department of Medicine, University of North Carolina, Chapel Hill, NC USA; 4 Group Practice and Centre for Allergy, Respiratory and Sleep Medicine, Red Cross Maingau Hospital, Frankfurt, Germany; 5 Division of Respiratory Medicine, Women’s College Hospital, University of Toronto, Toronto, ON Canada; 6 Centre of Academic Primary Care, University of Aberdeen, Aberdeen, UK; 7 Observational and Pragmatic Research Institute, Singapore, Singapore

**Keywords:** Bronchodilation, Chronic obstructive pulmonary disease, Combination bronchodilator agents, Inhalers, LABA, LAMA, Pharmacotherapy

## Abstract

Inhaled fixed-dose combinations (FDCs) of a long-acting β-agonist (LABA) and a long-acting muscarinic antagonist (LAMA) have become the cornerstone for the maintenance treatment of symptomatic COPD patients. In this regard, global COPD treatment guidelines have recognized the importance of inhaler devices as integral contributors to the effectiveness of LABA/LAMA FDCs and recommend regular assessment of inhaler device use by the patients in order to improve long-term clinical outcomes. Optimal disease control is also highly dependent upon patient preferences and adherence to inhaler devices. This review objectively examines and compares the major inhaler devices used to deliver different LABA/LAMA FDCs, discusses the inhaler device characteristics that determine drug deposition in the airways, real-life preference for inhaler devices, and handling of inhaler devices that impact the results of the long-term management of COPD. The introduction of new LABA/LAMA FDCs, new inhaler devices, and more clinical studies have created confusion among physicians in choosing the optimal inhaled therapy for COPD patients; in this context, this review attempts to provide an evidence-based framework for informed decision-making with a particular focus on the inhaler devices.

**Funding.** The preparation of this manuscript was funded by Novartis Pharma AG.

## Introduction

Chronic obstructive pulmonary disease (COPD) is an increasingly common respiratory disease caused by substantial long-term exposure to noxious particles or gases and marked by persistent respiratory symptoms and airflow limitation [1]. COPD affects an estimated 210 million people worldwide [2]. By 2030, COPD is projected to be the third leading cause of mortality globally [3]. The significant economic burden imposed by COPD continues to increase both in terms of direct and indirect healthcare costs [4, 5].

The Global Initiative for Chronic Obstructive Lung Disease (GOLD) report provides a strategy for the assessment and management of COPD and suggests categorizing these patients into four groups, A–D, based on symptoms and exacerbation history [6]. Inhaled therapy is fundamental in all classes of COPD patients. GOLD recommends the combination of a long-acting β-agonist (LABA) and a long-acting muscarinic antagonist (LAMA) as the first-line treatment for patients in GOLD groups B and D, i.e., patients with high symptom burden and those who are at a greater risk of exacerbations, respectively. Starting with LABA/LAMA combination therapy is recommended on the basis of the greater efficacy of this therapy in improving lung function, symptoms, quality of life, and in reducing exacerbations when compared to monotherapy or to LABA/inhaled corticosteroid (ICS) combinations in these patients [6–15].

The current GOLD strategy has explicitly recognized the importance of inhaler choice and instructions in the context of COPD management. Moreover, for the first time, GOLD 2019 has recommended to consider switching molecules and/or inhaler devices within classes to improve response/outcomes [6]. Following careful device selection tailored to individual patient needs and abilities, the importance of initial education and training in inhaler device technique is emphasized. Regular reassessment of inhaler technique has been recommended to improve long-term therapeutic outcomes. Finally, before concluding that the current treatment is insufficient, inhaler technique (and adherence to therapy) should be reviewed [6]. The GOLD strategy has also recognized the importance of delivering more than one drug via a single inhaler device, especially in light of the evidence that use of multiple devices requiring different inhalation techniques diminishes the effectiveness of therapy [16]. Long known to being a critical issue in asthma management, ensuring adequate inhalation technique may be of even greater importance in older COPD patients who are more likely to have debilitating comorbidities such as arthritis of the hands and typically have far less ventilator reserve [17, 18].

Fixed-dose combinations (FDCs) of LABA/LAMA have become the foundation of COPD treatment and this article provides an overview of the key aspects of inhaler devices that are used to deliver this therapy in FDCs to patients with COPD. More importantly, this review is aimed to provide guidance to physicians on evaluating device characteristics and ensuring correct inhaler use by patients, in light of the renewed focus on patients’ ability to use these devices correctly for optimal treatment outcomes. We describe key inhaler device-related factors that influence the patients’ and physicians’ perception of devices, ultimately impacting the effectiveness of LABA/LAMA combination therapy in COPD.

### Compliance with Ethics Guidelines

This article is based on previously conducted studies and does not contain any studies with human participants or animals performed by any of the authors.

## Delivery of LABA/LAMA Combination Therapy to COPD Patients

Several devices are available to deliver LABA/LAMA in a FDC to COPD patients and each device has its own features that should be considered when tailoring treatment to specific patient needs. These devices include pressurized metered-dose inhalers (pMDIs), dry powder inhalers (DPIs), and soft-mist inhaler (SMI) [19] and are listed in Table 1.

**Table 1:** Key characteristics of the inhaler devices used for LABA/LAMA delivery

Inhaler type	Formulation	Available devices	LABA/LAMA medications delivered	Advantages	Limitations
Pressurized metered-dose inhaler	Drug suspended or dissolved in a propellant	Aerosphere^®^	Formoterol and glycopyrronium	Compact and portable Offer consistent dosing and rapid delivery Can be used independently and unobtrusively For many COPD patients, it is possible to easily achieve the slow inhalation flow required with a pMDI with training When used with a valved holding chamber, improvement in lung deposition of drug particles and reduction in hand–breath (activation–inhalation) coordination problems is seen	Patients with poor dexterity or weak grip may find it difficult to actuate the device Actuation before inhalation is common Failure of proper hand–inhalation coordination while using a pMDI results in greatly reduced doses of drug reaching the lungs Lack feedback mechanisms confirming dose delivery Contain propellants (required to generate the aerosol cloud and also for suspension or dissolution of active ingredient) Patients would not breathe out to empty lungs before inhalation (due to lack of proper perception of airflow resistance) Patient’s head should always be tilted back for proper inhalation
Dry powder inhaler	Drug blended in lactose; drug alone; drug/excipient particles	Breezhaler^®^ Neohaler^®^ Ellipta^®^ Genuair^®^	Indacaterol and glycopyrronium; vilanterol and umeclidinium; formoterol and aclidinium	Compact and portable Breath-actuated: do not require coordination of inhalation with activation and do not require hand strength Some DPIs have a feedback mechanism for the patient to ensure they have inhaled the medication Do not contain a propellant	Requires a minimum inspiratory flow, which is related to the device’s resistance and varies from one device to another Geriatric and/or patients with very severe COPD may lack the ability to generate sufficiently high inspiratory flows through some DPIs, therefore compromising, if not preventing, dose delivery Most inhalers are moisture sensitive Patients would not breathe out to empty lungs before inhalation (due to lack of proper perception of airflow resistance) Patient’s head should always be tilted back for proper inhalation
Soft-mist inhaler	Aqueous solution	Respimat^®^	Olodaterol and tiotropium	Portable Multi-dose device The relatively long generation time of the aerosol could facilitate coordination of inhalation and actuation Does not contain a propellant	The dispensed metered volume per dose of 15 µL limits the dose-delivery capacity to drugs with adequate solubility with respect to the required dose Requires hand–breath coordination All patients may not be able to independently load the cartridge in the device chamber prior to initial use or to activate the device in between doses (turning lever-dexterity issues) Two actuations are required to achieve delivery of the daily treatment dose

The pMDIs are widely used because of their small size and unobtrusive nature. Their use continues despite evidence of frequent coordination errors and mishandling of these devices by patients [20–23]. However, the CRITIKAL study showed that poor coordination between the start of an inhalation and actuation of the dose (i.e., actuation coming before inhalation) was a critical error with MDIs that was associated with poor disease outcomes [24]. The CRITIKAL study results also indicated that exhaling into the mouthpiece or not holding the inhaler upright was a critical pMDI error; moreover, inspiratory effort was not slow and deep enough in the majority of asthma patients using a pMDI [24]. Lack of device knowledge, incorrect second dose preparation, timing, or inhalation, exhaling into the mouthpiece, and not holding the inhaler upright have also been identified as critical errors associated with pMDIs [24–26]. A notable development has been breath-activated pMDIs, which incorporate a triggering mechanism that releases the dose when a patient’s inspiratory effort is detected [21, 22]. The use of a valved holding chamber (a reservoir with a one-way valve permitting airflow into the patient’s mouth) to activate the pMDI before inhalation has been propagated to eliminate potentially critical inhaler handling errors and to increase lung deposition of drug particles [27, 28].

The DPIs are devices containing drugs in powdered formulation consisting of micronized particles in a respirable range [29]. Most DPIs allow the particles to be deagglomerated using energy created by the patient’s own inspiratory flow. These devices are available as single- and multiple-dose configurations [29, 30]. DPIs are breath-actuated and thus they do not have the issue of coordinating actuation and inhalation [31]. DPIs offer increased stability of drug formulation, flexibility in inhaler design options, and ability to achieve a high fine particle fraction [32]. However, DPIs do have some inherent limitations, e.g., variable airflow resistance, and often the inability of patients to achieve adequate inspiratory flow in order to mobilize the dry powder medication. However, some patients fail to generate sufficient inspiratory effort even if they are capable of achieving it [33]. The CRITIKAL study showed that insufficient inspiratory effort was a critical error associated with the use of DPIs [24]. Although DPIs obviate the issue of coordinating inhalation to an actuation, other errors are seen such as incorrect loading and preparation of the dose, blowing into the device, and exposing multi-dose reservoir devices to environmental moisture [24, 29, 33].

The SMI is a multiple-dose, propellant-free, hand-held, liquid inhaler device that generates an inhalable aerosol from a drug solution using a patient-independent and reproducible energy supply [34]. The aerosol plume generated by this device is slower and lasts longer than aerosol clouds from pMDIs [35]. Limitations of the SMI include potential issues in dose preparation, the device being non-breath-actuated, unavailability in many countries, and relatively higher costs compared with other devices [36].

In the context of the limitations and advantages of different classes of devices (Table 1), the shift from pMDIs to DPIs and SMI signifies a development in inhaled therapy. The newer inhaler devices exclude propellants, minimize patient limitations (including cognitive and psychomotor impairment that may limit inhaler use) and errors in handling the device, and improve the consistency of drug delivery to the lungs. In the subsequent sections, we describe representative devices from these classes of inhalers.

## Inhaler Devices Available for LABA/LAMA Delivery

### Aerosphere^®^

The Aerosphere^®^ (AstraZeneca Pharmaceuticals, Wilmington, DE, USA) is a hydrofluoroalkane-propelled pMDI containing 20–180 inhalations [37]. The canister has an attached dose indicator and is supplied with an actuator body and mouthpiece with a cap. Aerosphere^®^ contains porous particles that form a co-suspension with drug crystals; the porous particles are comprised of the phospholipid 1,2-distearoyl-*sn*-glycero-3-phosphocholine and calcium chloride. After each actuation, the device delivers glycopyrronium 7.2 µg and formoterol furoate 4.8 µg from the actuator. Priming the Aerosphere^®^ before the first dose is essential to ensure appropriate drug content in each actuation; priming before first use requires four sprays (actuations) into the air away from the face, shaking well prior to each spray [37].

### Breezhaler^®^

The Breezhaler^®^ (Novartis Pharma AG, Basel, Switzerland) is a breath-actuated, single-dose, capsule-based DPI used to deliver a variety of medications, including indacaterol (a LABA), glycopyrronium (a LAMA), indacaterol/glycopyrronium FDC and budesonide (an ICS) [38]. In case of Ultibro^®^ Breezhaler^®^, each delivered dose contains 110 μg of indacaterol maleate equivalent to 85 μg of indacaterol and 54 μg of glycopyrronium bromide equivalent to 43 μg of glycopyrronium [38]. The Breezhaler^®^ requires the loading of a drug-containing capsule prior to each inhalation. Generally, one inhalation is enough to empty the capsule for most patients. Should the capsule not completely empty upon a shallow and short inspiration, e.g., low inhaled volume, patients will see powder remaining in the capsule and can therefore repeat the inhalation manoeuver. The Breezhaler^®^ was designed to provide immediate sensory feedback to the patient that the dose has been administered correctly: by hearing a distinctive “whirring” noise on correct inhalation, by visually checking that the transparent drug capsule is empty, and by tasting the lactose excipient [38]. The Breezhaler^®^ has a low intrinsic resistance; most patients are able to generate the minimum inspiratory flow rate of 30 L/min with Breezhaler^®^ and the device provides consistent dose delivery using inspiratory flow rates between 30 and 100 L/min [39–41]. Low-resistance devices such as Breezhaler^®^ allow air to flow through them easily [41]. Owing to its low resistance, Breezhaler^®^ provided consistent dose delivery with regards to both the delivered dose and fine particle mass across the range of inhalation flow rates achievable by COPD patients [42, 43]. Patients with mild to very severe COPD have been shown to use the Breezhaler^®^ device successfully, with a low device complaint rate (< 0.03%) and no device failures from approximately 90,000 recorded uses [38]. The Breezhaler^®^ was shown to deliver a higher fine particle fraction and greater drug deposition in the lungs (lower oropharyngeal drug deposition) compared with the high-resistance HandiHaler^®^ DPI [44]. Multiple steps are required for drug administration with Breezhaler^®^, which may induce errors; regardless of this, the recent large-scale real-world INHALER study showed that patients committed fewest errors with Breezhaler^®^ versus any other studied inhaler, including pMDIs and SMIs [45].

### Ellipta^®^

The Ellipta^®^ DPI (GSK, Research Triangle Park, NC, USA) is single-step activation, multiple-dose inhaler that comes in a two-strip configuration for delivery of LABA/LAMA combination [46]. It was designed to deliver LABA/LAMA dual bronchodilator FDC such as vilanterol and umeclidinium [47]. Anoro^®^ Ellipta^®^ delivers 55 μg of umeclidinium and 22 μg of vilanterol per dose. Compared with other DPIs, the Ellipta^®^ device requires fewer steps for actuation and use requiring only that the patient open the mouthpiece cover fully, inhale the powder, and close the mouthpiece [46, 47]. Ellipta^®^ has a medium airflow resistance; in vitro data showed that doses of drugs delivered via the Ellipta^®^ device were consistent at inspiratory flow rates of at least 30 L/min [31, 48]. This suggests that Ellipta^®^ can be used even by patients with severe COPD notwithstanding that real-life use may differ from that observed in randomized controlled trials. A frequent error of insufficient inhalation effort observed with Diskus^®^ in the CRITIKAL study has also been observed with Ellipta^®^. In an in vitro study that replicated inhaler-specific patient inhalation profiles that were previously recorded in vivo using the Electronic Lung (eLung™), drug dose delivery via the Ellipta^®^ DPI was consistent across the range of patient representative inhalation parameters for all therapies such as formoterol/vilanterol, umeclidinium/vilanterol, and formoterol [49]. A recent study showed that patients with mild to very severe COPD could also generate sufficient inspiratory flows for optimum drug delivery via Ellipta^®^ [50]; it should be noted that this may not always be true in real life.

### Genuair^®^

Genuair^®^ (AstraZeneca, Cambridge, UK) is a multi-dose DPI designed to deliver inhaled medications such as FDC of formoterol and aclidinium to patients with COPD [51, 52]. Each delivered dose contains 396 μg of aclidinium bromide (equivalent to 340 μg of aclidinium) and 11.8 μg of formoterol fumarate dihydrate. The device is relatively easy to use: the patient need only remove the cap on the mouthpiece, press and release the green button at the back; with successful inhalation the color of the control window turns from green to red with an audible click [52]. Genuair^®^ has a medium resistance to inspiratory airflow and uses an optimized dispersion system to ensure effective deagglomeration of the inhalation powder [52, 53]. Genuair^®^ has been shown to deliver a consistent fine particle dose at inspiratory flow rates of greater than 35 L/min [53, 54]. The device provides a fine particle fraction averaging 40% [55]. A limitation for Genuair^®^ is the initial flow acceleration which needs high effort.

### Respimat^®^

Respimat^®^ (Boehringer Ingelheim, Ingelheim, Germany) is a multi-dose, propellant-free, hand-held SMI that delivers FDC of olodaterol and tiotropium [56]. The delivered dose is 2.5 μg tiotropium (as bromide monohydrate) and 2.5 μg olodaterol (as hydrochloride) per puff. The device works by forcing a metered dose of the drug solution through a precisely engineered nozzle, producing two fine jets of liquid that converge at a preset angle; this generates an aerosol cloud (the soft mist) [57]. The aerosol spray exits the Respimat^®^ more slowly and for a longer duration than with the pMDIs, resulting in a higher fraction of fine particles than most pMDIs and DPIs. This translates into lower oropharyngeal deposition and consequently higher lung drug deposition, higher than with a pMDI [34, 57]. In clinical trials in patients with COPD, bronchodilator drugs delivered from Respimat^®^ were equally as effective in bronchodilation at half the dose delivered from a pMDI and 3.6 times more effective than the Handihaler^®^ DPI [57]. Respimat^®^ was consistently shown to be well accepted by COPD patients, largely because of its inhalation and handling characteristics [57]. As the metered volume is fixed at 15 μL, Respimat^®^ is limited to drugs with adequate solubility in order to deliver the required dose [25, 56]. Additionally, the patients need to have good dexterity to twist and open the cap.

## Characteristics of LABA/LAMA Inhaler Devices: Drug Deposition and Airflow Resistance

The most important factors that determine drug deposition in the airways through inhalation include device characteristics, type of drug formulation, deagglomeration, particle size, oral and bronchial deposition, aerosol physical properties (e.g., aerosol velocity), and patient characteristics (such as inspiratory flow, disease state, preparation of the device, coordination of steps) [20, 21]. These factors ultimately determine patients’ functional and clinical responses to the treatment. Key device attributes pertinent to inhaler choice and patient adherence include convenience, ease of use, simple instructions, minimal potential for errors, airflow resistance, efficiency of delivery, and cost [58]. Consequent to the technological advances in the design of inhaler devices, the newer inhaler devices afford a pulmonary drug deposition fraction of 30–50% of the nominal dose (Table 2), substantially higher than the 10–15% with older devices [33].

**Table 2:**
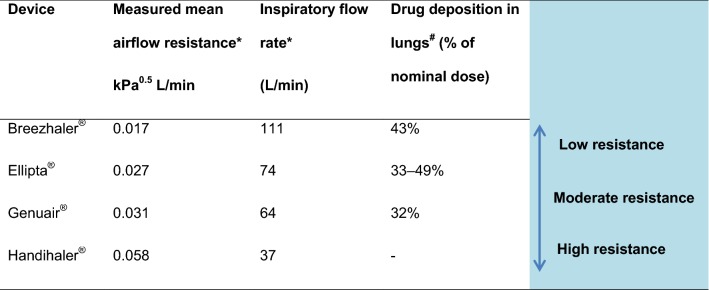
Device intrinsic airflow resistance influences the inspiratory flow rate that patients can achieve [59] and drug deposition in lungs with different DPI inhaler devices

*The pressure drop and corresponding flow rate were measured at a defined pressure point or a constant flow rate (0–100 L/min) using a test system with a mass flow meter, a differential pressure sensor connected to a sampling tube, a flow control valve, and vacuum pumps. Inspiratory flow resistance was calculated by linear regression using the method of least-squares

^#^Combining in vitro mouth–throat deposition measurements, cascade impactor data, and computational fluid dynamics simulations

The turbulent force generated by the patient is responsible for deaggregation of the powder into smaller particles and hence for the available amount of optimum-sized particles for drug deposition. This energy is the product of the patient’s inspiratory flow and the device’s intrinsic resistance [32, 59]. As Table 2 shows, with the low-resistance Breezhaler^®^ a much higher inspiratory flow is generated while achieving adequate deposition than that achieved with a higher intrinsic resistance. In this context, the intrinsic resistance of a DPI device refers to the inspiratory flow rate required to release the correct amount of drug. Accordingly, within the DPI class, there are high (required inspiratory flow rate 90 L/min), medium (60–90 L/min), and low (< 50 L/min) intrinsic resistance devices [30, 36]. The lower the device’s intrinsic resistance, the smaller the effort required from the patient to generate such airflow, which may be especially important in patients with severe airflow limitation. However, perturbations in expiratory airflow are not necessarily predictive of impaired inspiratory flow rates [60]. Among the DPIs that deliver LABA/LAMA, Breezhaler^®^ has the lowest airflow resistance, followed by Ellipta^®^ and Genuair^®^ [61] (Table 2). Moreover, a recent study showed that patients with COPD were able to inhale with the least inspiratory effort and generate the highest mean PIF via the Breezhaler^®^ inhaler than with the Ellipta^®^ and HandiHaler^®^ inhalers, irrespective of patients’ COPD severity, age, or gender [62]. Low resistance characteristics could explain patient-reported “comfort” inhaling through some devices; the flow rate produced by a standard pressure drop of 4 kPa was greater using Breezhaler^®^ than other DPI devices in vitro [63]. It is important to note that for DPIs, the speed of particles upon ejection from the mouthpiece, the disaggregation of the drug, the distribution of drug within the lungs, and the variability of the effective inhaled dose are optimal only when the inhalation flow rate and the intrinsic resistance of the device are balanced [32]. For example, a device such as Genuair^®^ with low variability in the aerodynamic characteristics and medium intrinsic resistance was shown to combine the positive aspects of achievable flow rates, consistent and efficient fine particle generation, and reduced impaction losses in the upper airways [59]. An in vitro comparison of four LABA/LAMA inhaler devices through modeling of the lung deposition showed that the Respimat^®^, an SMI device, provided the lowest amount of particles deposited in the mouth–throat region and the highest amount reaching all regions of the simulation lung model, followed by the DPI devices Breezhaler^®^, Ellipta^®^, and Genuair^®^ [63].

Patient demographics and clinical characteristics may also influence drug delivery; an assessment of inhalation characteristics showed that adults with asthma had greater inspiratory capacity than patients with COPD but children with asthma had the least capacity [64]. There is still ambiguity with regards to the effect of intrinsic resistance on drug deposition. A higher inspiratory rate with a high intrinsic resistance device would result in a higher particle deagglomeration, but a higher airflow rate would also lead to increased drug particle velocity, which is expected to result in higher oropharyngeal drug deposition. The behavior of the upper airway may have an impact on drug deposition; in this regard, studies have assessed how the human upper airway behaves with different resistances and geometries of the inhalers and in turn affects drug deposition [65]. For high-resistance inhalers, a correlation between maximum inspiratory pressure (MIP) and change in airway volume was shown with those exhibiting expansion in the upper airway having generally low MIP [66]; a linear relationship was observed between airway volume changes and maximum calculated volumetric airflow [66]; evaluation of the impact of inhalation maneuvers, inhaler mouthpiece geometries, and a stepped mouthpiece on the size of the upper airway showed that enlarged size of the upper airway might decrease aerosol deposition in the upper airway and increase lung deposition [67]. Additionally, a high inspiratory flow rate may be difficult to attain in children, elderly, and in patients with severe airflow obstruction in COPD. Lower peak inspiratory flow rates generated from a DPI, measured using an In-Check DIAL device, were observed in patients with COPD or asthma who were older than 60 years, compared with younger patients [68]. That said, clinical studies have shown that most patients were able to use a high-resistance DPI effectively, even during exacerbations [55, 60]. Consequently, it has been suggested that peak inspiratory flow rates should be measured prior to discharge of patients admitted for acute COPD exacerbation and during clinic visits to ensure optimum device selection and drug delivery of COPD patients, especially in the elderly, female patients, and those with short stature [69]. However, we should also consider that achieving a specific flow rate is also dependent on the resistance of the device; in this context, high inspiratory flow rate alone may not be enough to test the patient’s ability to use a distinct device. Hence, we suggest that the patient should be tested on his prescribed device. Despite the lack of complete understanding of the relationship between drug deposition and airflow resistance, DPIs appear to be suitable devices to deliver inhaled medications to patients with COPD of varying severity. Nevertheless, it is important to consider that patients with very severe COPD were not systematically included in all clinical studies, and as an example patients who are undergoing acute exacerbations of COPD, i.e., a hospitalization, are typically not enrolled in any study, therefore making it difficult to evaluate the appropriateness of different types of inhalers.

Ultimately, healthcare professionals and caregivers should appreciate that on the basis of the clinical data showing significant efficacy throughout a wide spectrum of disease severity, all devices appear to be adequate for use regardless of their different physical properties. Thus, it is worthy to consider that clinical outcomes throughout randomized controlled studies or in real-life settings may not always be affected by physical characteristics or theoretical issues. In other words, one cannot dissociate the overall clinical efficacy of inhaled medications for COPD from inhaler specificities and handling aspects within the context where evidence was generated.

## Preference for LABA/LAMA Inhaler Devices

Patients’ and physicians’ preference for a particular inhaler device influences treatment adherence in the long-term management of COPD that in turn affects the treatment outcomes.

In a large real-word study that assessed inhaler preference in patients with COPD, the ease of use, dose delivery recording (delivery feedback), and dose capacity (single- or multi-dose) were cited by patients as the most important device attributes while choosing a device. Moreover, key factors that patients considered made the device easier to use were fewer steps to operate the inhaler, easier coordination of breathing manoeuver, and least resistance while inhaling [58]. For healthcare providers, patient satisfaction and ease of use were considered as the most important attributes when selecting an inhaler device for patients [58]. Another real-life study assessed and compared the patients’ preference for Breezhaler^®^, Genuair^®^, and Respimat^®^ in asthma and COPD outpatients by means of a device handling questionnaire [70]. In this study, Genuair^®^ and Respimat^®^ were the most liked and were perceived by patients as the easiest to use. Patients and nurses also perceived these two devices as the least problematic; it should be noted that in this study, patients were not asked to insert the cartridge into the Respimat^®^ device, which remains a vital step prior to prepare the actuations [70]. Mean number of attempts required to achieve the first effective actuation was the highest with the Breezhaler^®^ device, therefore reinforcing the importance of patient education with a new device. Respimat^®^ proved to be the most preferred in COPD patients since it was the most liked and its success rate at first attempt was the highest. Furthermore, previous experience with DPIs and/or MDIs did not affect preference for an individual device in patients with COPD or asthma [71]. Respimat^®^ was preferred over the pMDI by patients with COPD and other obstructive lung diseases [71]. In comparative studies with pMDIs, the patient total satisfaction score with Respimat^®^ was statistically and clinically significantly higher than with the comparator pMDI [71]. In a cross-sectional study among patients with COPD in Spain using the validated Patient Satisfaction and Preference Questionnaire (PASAPQ), patients reported satisfaction with both Respimat^®^ and Breezhaler^®^ devices [72].

Several studies were previously carried out to compare ease of use and patient satisfaction with commercially available inhalers. While all devices tested in a non-interventional setting were deemed acceptable to patients, statistically significant differences were nevertheless observed in the questionnaire ratings from different inhalers [73]. Notably, patients with severe COPD expressed a higher feeling of satisfaction with their devices than those with moderate or mild disease, independent of the device used; this may have been due to the longer use, familiarity with the device, and probably better adherence (because of their severe symptoms) of patients with severe COPD [73]. In DPI-naïve patients with COPD, Breezhaler^®^ was preferred over HandiHaler^®^ and was more likely to be used correctly [74]. A real-life study evaluated inhaler preference and handling errors with the Ellipta^®^ and Breezhaler^®^ DPIs in device-naïve Japanese volunteers aged 40 years or older [75]. It was observed that Ellipta^®^ DPI was preferred over Breezhaler^®^ on the basis of its ease-of-use features and was associated with fewer handling errors [75]. In contrast, in the ADVANTAGE study, device-naïve patients with COPD reported greater preference for the Breezhaler^®^ than for the Ellipta^®^ device for confidence of dose delivery and comfort of the mouthpiece [76]. Such differences may be linked to the larger mouthpiece of the Breezhaler^®^ as well as to the ability for patients to visually confirm if any powder is left in the capsule after each actuation. These results were confirmed in the recent Real life Experience and Accuracy of inhaLer use (REAL) survey conducted in patients with COPD, which gathered insights into real-life inhaler use by patients and healthcare providers, device attributes, and training [77]. The majority of patients using Breezhaler^®^ reported either being very confident or confident of having taken a full dose, which was higher than those using Genuair^®^, Ellipta^®^, and Respimat^®^. However, this study also identified a low incidence of patient training and monitoring by healthcare providers for correct inhaler use [77]. In the recently reported INHALATOR study, a significantly greater proportion of patients expressed preference for Breezhaler^®^ than for Respimat^®^ (57.1% versus 30.1%) [78]. It should be noted that good clinical practice implies that patients are educated on how to use a new device upon first encounter, while the INHALATOR study design was apparently relying on patients familiarizing themselves with the new device via product leaflets; also, the study was meant to evaluate the correct use and satisfaction rather than the efficacy between devices [78]. It is important to note that differences in active drug in each of the devices evaluated, among other limitations in the INHALATOR study, may have influenced the overall results.

It is noteworthy that in the inhaler device preference studies that were sponsored by the pharmaceutical companies, the sponsor’s device seemed favored by the type of questions asked to participants and typically came out as a preferred choice in most of the studies. Unsurprisingly, these observations suggest an intent to highlight differential device specificities. Patients with unstable disease or who were unable to use inhalers were usually excluded and the extent of instruction and coaching given in the studies was highly variable. Interestingly, some studies sponsored by pharmaceutical companies found no significant differences in terms of patient satisfaction between different types of devices (including DPIs and SMI) [58, 70, 79]. On the other hand, and as discussed in the following section of this review, studies including CRITIKAL [24] and INHALER [45] showed different specific errors associated with different inhaler devices. These studies, along with a recent systematic review, demonstrated the impact of critical errors in handling inhaler devices on health outcomes in patients with COPD and asthma [80].

There have been no well-designed studies that attempted to evaluate how differences among the devices would translate in terms of relevant patient outcomes. Moreover, patient preference is as important as clinical evidence when selecting an appropriate device and ultimately in realizing optimal clinical outcomes; therefore, improved patient education, patient–physician interaction, and affordability along with greater ease of using inhaler devices would lead to correct inhaler choice.

## Handling of Inhaler Devices

In randomized clinical studies that compare inhaled treatments in COPD, the correct use of inhaler devices is an inclusion criterion. However, in real life, patients continue to make errors with their usual inhaler device [81], which may negate the treatment benefits observed in clinical studies. In this regard, various studies have assessed inhaler handling in real life. It has been shown that most inhaler users not only make errors but also those patients who did not get proper education on inhaler technique are more inclined to misuse their device [82]. A recent Japanese study has also suggested that patients, regardless of having asthma or COPD, require to be instructed at least three times, i.e., given demonstrations by trained personnel in order to limit inhaler handling errors [83]. Notably, while about 65% of patients made at least one handling error that could affect the efficacy after an initial guidance on how to use the Breezhaler^®^, the Ellipta^®^ or Respimat^®^, more than 90% of patients using any device could successfully learn the correct use after receiving guidance from pharmacists three times successively [83]. Whether inhaler handling errors remain frequent among long-term inhaler users or are associated with worse clinical outcomes in COPD is discussed below.

The real-life INHALER study assessed inhaler device handling in approximately 3000 COPD patients who were using inhaler devices for at least 1 month [45]. Physicians assessed patients’ inhaler technique and documented device-dependent (i.e., specific) or device-independent handling errors. Handling errors were observed in over 50% of inhalations regardless of the device used. However, the number of errors deemed critical (i.e., those which significantly reduced drug delivery) differed amongst the devices. Device-independent errors (e.g., patient did not exhale fully prior to inhalation) were equally frequent across all devices. Fewer patients made critical errors using Breezhaler^®^ versus other devices; Breezhaler^®^ also had fewer device-specific errors. Overall, fewer critical errors were made with DPIs than with pMDIs or SMIs. Moreover, the recent INHALATOR study showed that the rate of correct device use, i.e., no critical errors during inhalation technique, was similar between Breezhaler^®^ and Respimat^®^. The evaluation of the patients’ inhalation technique was based on the investigator’s observation [78].

**Fig. 1:**
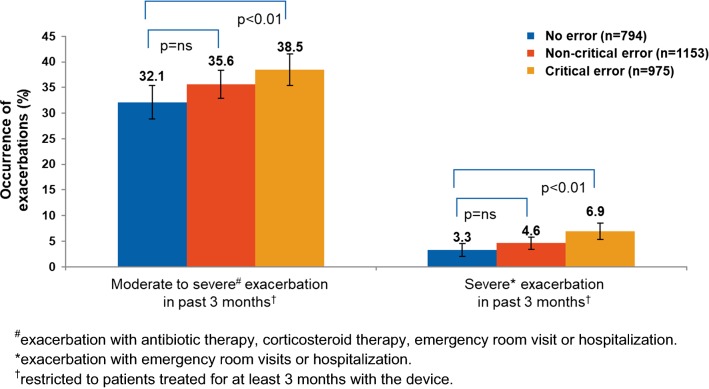
Association of critical device handling errors with COPD exacerbations

An important finding of the aforementioned INHALER study was that the handling errors were significantly associated with more frequent COPD exacerbations (Fig. 1) [45].

Use of Genuair^®^ was associated with fewer errors compared with HandiHaler^®^, including critical errors that may impede the delivery of sufficient doses or drug deposition to the lungs [52, 54]. In an assessment of critical inhaler technique errors with Genuair^®^ and Breezhaler^®^ after 2 weeks of daily use, the proportion of patients making these errors was low with both Genuair^®^ and Breezhaler^®^ [84]. When compared with other inhalers, fewer COPD patients had at least one overall error using the Ellipta^®^ inhaler compared with the Handihaler^®^ or Breezhaler^®^; a larger proportion of patients rated the Ellipta^®^ inhaler very easy or easy to use compared with the Handihaler^®^ or the Breezhaler^®^ [85]. A cross-sectional study examined specific patient characteristics and device attributes that are associated with poor handling technique among patients with COPD who used at least one of the following devices: MDI, Diskus^®^, and Handihaler^®^ [86]. It was found that poor inhaler technique was common among individuals with COPD, varied amongst devices, and was even associated with race and level of education [86]. A real-life study compared handling of different inhaler devices (Aerolizer^®^, Autohaler^®^, Diskus^®^, or Turbuhaler^®^) in primary care practice in France and observed differences in device handling in primary care that were not considered in controlled studies [87]. Although this study did not compare devices that deliver LABA/LAMA FDCs, a comparison of findings from this study and those from the INHALER study [45] indicates that device handling has not improved significantly over the several decades that handling studies have been performed.

## Effective Treatment Through Appropriate Application of Inhaler Devices

The inhaled route of administration is considered as the best way to deliver medications to patients with COPD. The availability of an array of medication classes and associated inhaler devices with different degrees of efficacy has actually made the selection of optimal inhaled treatment complicated.

Ideally, inhalers should be easy to use and should have multiple feedback and control mechanisms that would reduce physician overestimation and ignorance of correct inhalation, allow compliance to be monitored, facilitate patient self-education, and give reassurance to patients in routine care. Treatment compliance in long-term disease management may be improved by educating patients and physicians on the correct use of inhaler devices. In this regard, studies such as the REAL survey have attempted to assess the effectiveness of patient/healthcare provider training on correct inhaler use [77]. Poor inhalation technique, number of inhalation steps, clinical setting, and time elapsed since training were shown to have an impact on the effectiveness of the educational/training intervention [77]. Educational interventions to improve inhaler technique in patients were found to be effective in the short term [77]. To improve application of inhaler devices in real life, the German Airway League developed a checklist for inhaler devices to check for inhaler errors. Moreover, they prepared free internet-based short videos for all available inhaler devices. It was shown that a single session of patient information through a short video sequence improved device use, and the effect lasted for 4–8 weeks [88–90]. Video information seemed to be very important in improving inhalation technique, since healthcare personnel in primary care and hospitals are often not qualified for use of different inhaler devices [91, 92].

**Fig. 2:**
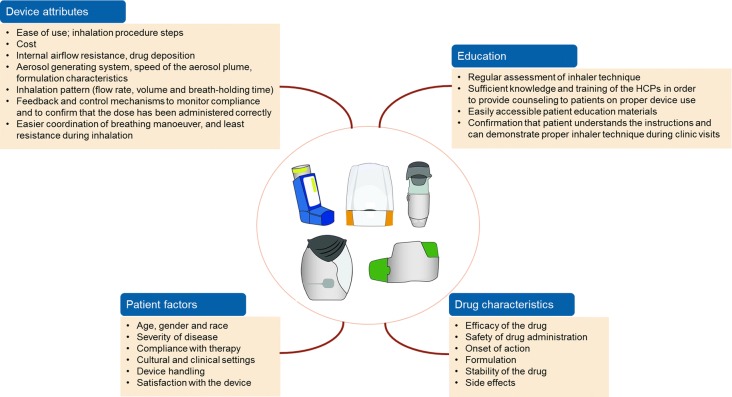
Factors influencing treatment outcomes from inhaler devices

A number of factors influence treatment outcomes with inhalation therapy (Fig. 2). In particular, characteristics of the drug and of the delivery device, patient’s ability to use a device properly, education/training, and patient’s personal preference should be considered in order to maximize treatment outcome through inhalation therapy [6, 93]. However, one should interpret device preference studies cautiously as they often focus on handling-related preferences and are frequently conducted with patients using placebo devices, therefore discarding some important aspects of inhaled medications. These include the patient’s ability to self-monitor adequate use of the device and uptake inhaled medication, as well as the clinical benefits of the inhaled therapy as perceived by the patients [94]. These factors have the potential to support patients’ adherence and satisfaction and deserve physicians’ consideration. Highlighting the association of inhaler device application with clinical outcomes, Molimard et al. showed for the first time that, despite limitations of their study (e.g., short follow-up period), inhaler misuse may be linked to increased rates of severe exacerbations in COPD patients [45].

As inhaled medications are essentially “integrated” with their respective devices, the challenge in ensuring the best possible application of inhaled treatment is in identifying whether the empirical clinical efficacy of the delivered molecules or the differential inhaler use (more/less clinical errors, or variable patient behaviors using different devices) determines the observed treatment efficacy. Even a randomized head-to-head comparison of different LABA/LAMA FDCs would be limited as the comparison is often made between different medications (even though of the same class) in different devices. A possible solution could be more head-to-head studies of different inhalers (e.g., administering placebo to remove medication bias or using a double-dummy design wherein all patients use all inhalers, but some will be placebo).

## Conclusions

Inhaler devices that offer consistent and efficient dosing, ease of use, and patient preference lead to enhanced patient adherence and therefore better treatment efficacy. Nonetheless, handling errors are common and numerous patient factors still limit the use of contemporary devices. Such suboptimal inhaler use has an adverse effect on clinical outcomes. The GOLD 2019 strategy has re-emphasized consideration of inhaler device attributes and handling while prescribing treatment to COPD patients. In this context, GOLD has explicitly stated that the importance of education and training in inhaler device technique cannot be overemphasized.

It is important that a patient’s ability to use an inhaler device is checked by the healthcare provider at the first visit and monitored at each subsequent visit, ideally every 3 months for a minimum of 1 year. Assessment of inhaler technique and adherence has been recognized by GOLD as an essential component of the management of stable COPD. Therefore, patient education and patient–healthcare provider interactions are key to ensuring the correct use of inhaler devices. Technologic advances may soon offer assistance. New electronic and internet-connected inhaler devices, also called smart inhalers, e.g., eBreezhaler^®^, are in late-phase development to help with real-time monitoring of treatment adherence and appropriate device use, and even to train patients. The widespread adoption of smart inhalers might be limited by concerns over cost-effectiveness, lack of evidence that they improve quality of life, and increased burden on healthcare providers to monitor the data [95].

Finally, in view of the gap that still remains in the selection and application of appropriate inhaler devices for delivery of optimal COPD treatment, this review indicates that both the efficacy of the drug and appropriate application of inhaler devices cannot be dissociated in the context of evolving COPD management that has placed an increasing emphasis on the use of LABA/LAMA fixed-dose bronchodilator combinations. Although influenced by physician and patient preferences, the choice of an appropriate inhaler and continuous educational efforts to reinforce appropriate device handling are of equal importance to ensure therapies optimally contribute to the management of COPD.
